# Whole genome mRNA expression profiling revealed multiple deregulated pathways in stromal vascular fraction from erectile dysfunction patients

**DOI:** 10.1042/BSR20181015

**Published:** 2018-11-23

**Authors:** Radhakrishnan Vishnubalaji, Muthurangan Manikandan, Abdullah Aldahmash, Abdullah AlJarbou, Mohamad Habous, Dulaim Alhajeri, Raed Almannie, Musaad Alfayez, Nehad M. Alajez, Saleh Binsaleh

**Affiliations:** 1 Stem Cell Unit, Department of Anatomy, College of Medicine, King Saud University, Riyadh 11461, Kingdom of Saudi Arabia; 2 Division of Urology, Department of Surgery, College of Medicine, King Saud University, Riyadh, Kingdom of Saudi Arabia; 3 Urology and Andrology Department, Elaj Medical Centers, Jeddah, Saudi Arabia; 4 Cancer Research Center, Qatar Biomedical Research Institute, Hamad Bin Khalifa University (HBKU), Qatar Foundation, Doha, Qatar

**Keywords:** Erectile Dysfunction, gene expression, stem cell

## Abstract

**Background:** Stem-cell-based therapies have recently been explored in the field of erectile dysfunction (ED). However, the cellular and molecular phenotype of adipose derived stem cells (ADSCs) stromal vascular fraction (SVF) from ED patients remains largely unknown. Herein we compared the global gene expression profile in the SVF from ED patients and healthy individuals and identified altered signaling pathways between the two groups.

**Methods:** Samples (2–5 g) of abdominal adipose tissue from ED patients (*n* = 6) and healthy individual controls (*n* = 3) undergoing elective cosmetic liposuction were collected. Immediately after removal, SVF was separated using Collagenase type I and type IV protocol. RNA was isolated and microarray experiments were conducted using the Agilent platform. Data were normalized and pathway analyses were performed using GeneSpring software.

**Results:** Our data revealed multiple differentially expressed genes between the ED and control group. Hierarchical clustering based on differentially expressed mRNAs revealed clear separation of the two groups. The distribution of the top enriched pathways for the up-regulated genes indicated enrichment in inflammatory response and T-cell receptor signaling, while pathway analysis performed on the down-regulated genes revealed enrichment in mitogen-activated protein kinase, TGF-β, senescence, FAK, adipogenesis, androgen receptor, and EGF–EGFR signaling pathways in SVF from ED patient.

**Conclusion:** Our data revealed the existence of multiple altered signaling pathways in the SVF from ED patients, which could potentially play a role in the etiology of this disease. Therefore, therapeutic strategies targeting these pathways might provide novel therapeutic opportunity for ED patients.

## Introduction

Erectile dysfunction (ED) is a common clinical disorder that affects primarily men older than 40 years of age [1]. Significant scientific developments throughout the past three decades have extended our indulgent of the physiology and pathophysiology of penile erection. However, in addition to the well-known causes of ED, other factors such as obesity, diabetes, hypertension, and numerous collective lifestyle factors, including lack of physical exercise, lower urinary tract symptoms, have also been linked to the development of ED [1–3].

Presently, the majority of ED patients are treated with phosphodiesterase type-5 inhibitors (PDE5is), such as sildenafil, tadalafil, vardenafil, and avanafil [4]. In addition, several other treatment options exist for ED, including lifestyle modifications and surgical intervention. With the exclusions of lifestyle modification and revascularization procedures, these methods just treat the indications of ED, proposing symptomatic assistance rather than a remedy for the underlying disease progression [5,6].

Therefore, a critical assessment of the contemporary state of knowledge is crucial to deliver perspective for future research and development of novel therapies. Recently, stem-cell-based therapies have gained attention as a potential substitute in the prevention of ED in various animal models. Interestingly, embryonic stem cells transduced with brain-derived neurotrophic factor (BDNF), adult bone marrow and adipose-derived stem cells were capable of stabilizing erectile function through intracavernous injection [7–10].

Preliminary clinical trials on postradical prostatectomy erectile dysfunction (pRP-ED) patients with bone marrow and adipose-derived stem cells have been reported with encouraging results [11,12].

However, the cellular and molecular phenotype of stem cells between control and ED patients remains largely unknown. Therefore, herein we compared the global messenger RNA (mRNA) expression profile in the SVF from ED patients and healthy individuals to identify the altered signaling pathways between the two groups.

## Methods

### Subjects

The study was approved by the Ethical Committee of King Khalid University Hospital and all the ED patients and healthy individual (control) involved gave informed consent. Samples (2–5 g) of abdominal adipose tissue obtained from abdominal subcutaneous adipose tissue (SAT) of six ED patients, aged from 29 to 68 and healthy individuals control, aged from 30 to 35 undergoing elective open-abdominal surgery (cosmetic liposuction) were collected (Table 1). None of the control individuals presented any chronic disease, diabetes, metabolic syndrome, or altered biochemical parameters that could indicate adipose tissue alterations. Immediately after removal, biopsies were washed in PBS and processed for the separation of SVF.

**Table 1:** Clinical characteristics for ED patients and healthy controls

Patient no.	Age	Co-morbid conditions	Body weight (kg)
**ED patients**			
1	29	ED, DM	68
2	47	ED, no other comorbidity	81.5
3	34	Left testicular cancer, postradical orchiectomy, ED	113.2
4	68	Prostate cancer, postradical prostatectomy, ED	76
5	53	DM, HTN, smoker, right renal cancer, postradical nephrectomy, ED	90.3
6	42	Hypothyroidism, primary infertility, ED	95
**Healthy controls**			
1	34	Smoker, bilateral varicoceles, no other medical problems, no ED	126.1
2	35	No other medical problems, no ED	86
3	30	No other medical problems, no ED	75

Abbreviations: DM, diabetes mellitus; ED, erectile dysfunctions; HTN, hypertension.

### Isolation of SVF

Stromal vascular fraction (SVF) isolation was carried out in accordance with our previously published protocols [13]. In brief, freshly isolated SAT biopsies and liposuction were collected in PBS and washed twice to eliminate peripheral blood. Next, samples were incubated in DMEM medium with 41% Collagenase type I and type IV (Gibco-Invitrogen, U.S.A.) for 45 min with gentle agitation at 37°C. After inactivation of collagenase by culture medium DMEM, undigested tissue was removed by filtering through a sterile 100 μm pore Cell Strainer (BD Falcon, CA, U.S.A.) and centrifuged at 600×***g*** for 10 min to separate the floating mature adipocyte layer from the pelleted SVF. SVF was resuspended in DMEM, filtered through a 40 μm pore Cell Strainer and centrifuged at 400×***g*** for 5 min. Pelleted SVF was resuspended in 500 μl of erythrocyte lysis buffer (RBC Lysis Solution, Puregene, MN, U.S.A.) and incubated for 3 min at room temperature. After centrifugation at 400×***g*** for 10 min, SVF was frozen in liquid nitrogen and stored at −80°C until RNA extraction.

### Gene expression microarray

RNA isolation and microarray analyses were carried out in accordance with our previously published protocols [14]. In brief, RNA was isolated using Total Tissue RNA Purification Kit from Norgen-Biotek Corp. (Thorold, ON, Canada) and were quantified using NanoDrop 2000 (Thermo Scientific, Wilmington, DE, U.S.A.). Total RNA was labeled and then hybridized to the Agilent Human SurePrint G3 Human GE 8 × 60k mRNA microarray chip (Agilent Technologies). All microarray experiments were conducted at the Microarray Core Facility (Stem Cell Unit, Department of Anatomy, King Saud University College of Medicine). Data were subsequently normalized and analyzed using GeneSpring 13.0 software (Agilent Technologies). Pathway analyses were conducted using the Single Experiment Pathway analysis feature in Gene Spring 13.0 (Agilent Technologies). Twofold cut-off with *P* < 0.02 was used. Target prediction was conducted using a built-in feature in Gene Spring 13.0 based on Target Scan database.

### Gene validation by qRT-PCR

Gene expression levels were validated in control and ED patients SVF cells. The procedure was performed in accordance with our previously published protocols [15]. In brief, SYBR Green-based qRT-PCR was performed using Applied Biosystems ViiA Detection system. 500 ng of total RNA was reverse transcribed using High Capacity cDNA Reverse Transcript Kit (Part No: 4368814; ABI) according to the manufacturer’s protocol. Relative levels of mRNA were determined from cDNA using real-time PCR (Applied Biosystems ViiA 7 Systems). Primer sequences used in the current study were VEGFA, PDGFRA, FOSB, JUN, IGF1, and LIF are listed in Table 2. The relative expression level was calculated using –ΔΔCT. GAPDH was used as an endogenous control.

**Table 2:** List of SYBR green primers used in current study

No.	Name	Sequence
1	GAPDH	5′ CTGGTAAAGTGGATATTGTTGCCAT
		5′ TGGAATCATATTGGAACATGTAAACC
2	VEGFA	TCACCAAGGCCAGCACATAG
		CGGCTTGTCACATTTTTCTTGTC
3	JUN	TGAGTGACCGCGACTTTTCA
		TTTCTCTAAGAGCGCACGCA
4	IGFI	5′ TCAGCAGTCTTCCAACCCAA
		5′ TGGTGTGCATCTTCACCTTCA
5	PDGFRA	5′ GACTAGTGCTTGGTCGGGTC
		5′ CAGGTTGGGACCGGCTTAAT
6	FOSB	5′ GCGCCGGGAACGAAATAAAC
		5′ CAACTGATCTGTCTCCGCCT
7	LIF	5′ GCCACCCATGTCACAACAAC
		5′ CCCCCTGGGCTGTGTAATAG
8	ZAP70	CCTGTACGTCCCCAGGTTTC
		CCGTAGAAGAAGGGCAGGTG

## Results

The present study composed of six ED patients, aged 29–68 and three healthy individuals; aged 30–35 undergoing elective open-abdominal surgery (cosmetic liposuction) were collected. Routine clinical evaluations were done for both groups including history, physical examination, basic lab testing, and IIEF questionnaire. The clinical characteristics of ED patients and healthy individuals included in current study are presented in Table 1. There was no significant difference in glucose, cholesterol, HDL, LDL, triglycerides, hemoglobin, and white blood count between the ED and control group (Supplementary Figure S1). ED patients had slightly lower LDL and platelets count compared with health controls (Supplementary Figure S1).

### Multiple dysregulated pathways in SVF from ED patients

**Figure 1:**
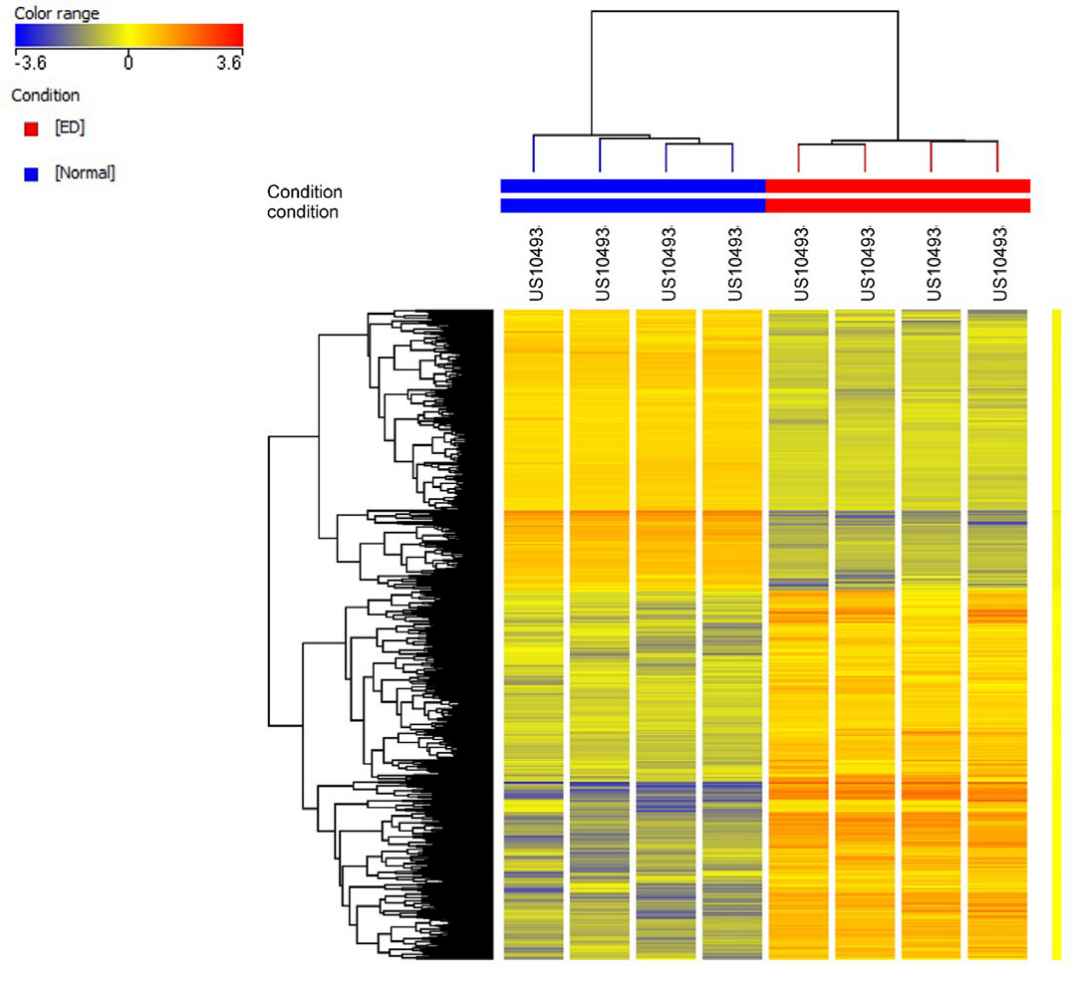
Differentially expressed genes in ED Hierarchical clustering of pooled ED and pooled healthy individual control samples based on differentially expressed mRNA levels. Each column represents a replica and each row represents a transcript. Expression level of each gene in a single sample is depicted according to the color scale.

**Figure 2:**
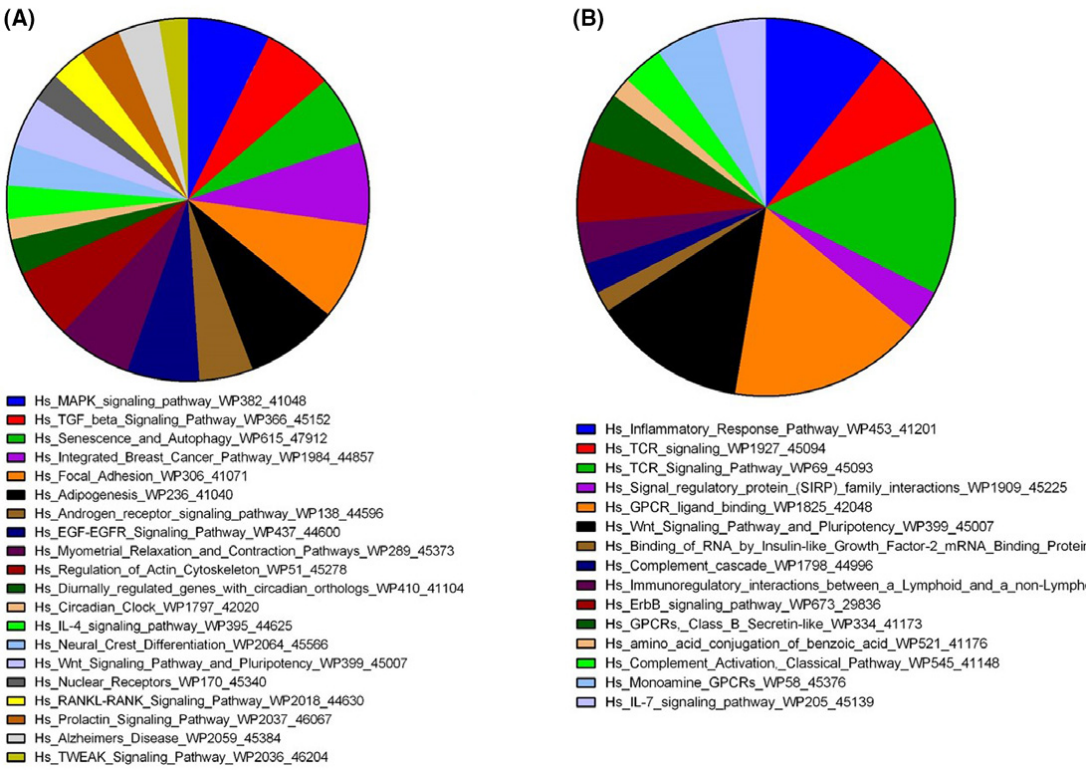
Pathway analysis of differentially expressed genes in ED patients (**A**) Pie chart illustrating the distribution of the top 20 pathway designations for the down-regulated genes in ED patients. (**B**) Pie chart illustrating the distribution of the top 15 pathway designations for the up-regulated genes in ED patients. The pie size corresponds to the number of matched entities.

**Figure 3:**
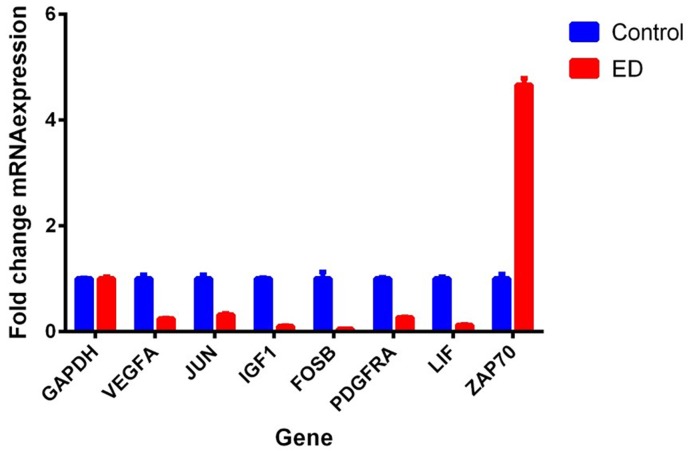
Validation of differentially expressed genes in ED using qRT-PCR Expression levels of selected genes from the microarray data with significant enrichment in the following pathways was validated using qRT-PCR, *n* = 3. **Angiogenesis:** VEGFA, PDGFRA; **FAK pathway**: PDGFRA, JUN; **TGF-β signaling:** FOSB, JUN; **Adipogenesis:** IGF1, LIF; **Inflammatory response:** ZAP70.

To understand the molecular alterations in SVF from ED patients, we executed global mRNA expression profile comparing SVF from ED patients with SVF obtained from healthy individuals (control). As shown in Figure 1, hierarchical clustering based on differentially expressed mRNAs revealed clear separation of the two groups, where multiple up-regulated and down-regulated transcripts in the ED patients were observed. The distribution of the top 15 enriched pathways for the up-regulated genes and top 20 enriched pathways for the down-regulated genes in SVF from ED patient are shown in Figure 2. Pathway analysis on the down-regulated genes revealed significant enrichment in several signaling pathways including mitogen-activated protein kinase (MAPK), TGF-β, focal adhesion, adipogenesis, androgen receptor, EGF–EGFR, regulation of actin cytoskeleton, circadian clock, IL-4, neural crest, Wnt, and RANKL-RANK, while inflammation and immune repose were the predominant pathways altered in the up-regulated gene in SVF from ED patients. Up (Inflammatory response: ZAP70) and down (Angiogenesis: VEGFA, PDGFRA; FAK pathway: PDGFRA, JUN; TGF-β signaling: FOSB, JUN; Adipogenesis: IGF1, LIF) regulated genes from the microarray data were subsequently validated using quantitative reverse transcription-PCR (qRT-PCR) (Figure 3). The FAK and angiogenesis signaling pathways are illustrated in Supplementary Figures S2 and S3.

Furthermore, our analysis on up-regulated genes revealed significant enrichment in numerous signaling pathways including inflammatory response, T-cell receptor (TCR), immunoregulatory, complement activation and IL-7. The inflammatory response signaling pathway is illustrated in Supplementary Figure S4.

## Discussion

**Figure 4:**
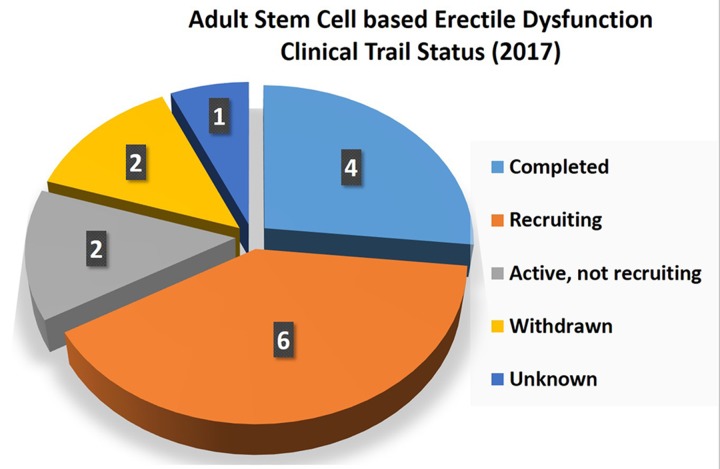
D pie chart elucidating the adult stem-cell-based clinical trial status for erectile dysfunction (2017)

Cumulative evidence in the literature suggested multi-lineage differentiation potential for adult stromal stem cells derived from different tissues [16,17], making them an attractive candidate for regenerative medicine. A number of stem-cell-based therapies for ED has recently investigated the therapeutic potential of transplanted adipose derived stem cells (ADSCs) or bone marrow stem cells (BMSCs) via intra-cavernous injection in animal models [7–10]. In humans, 15 studies were found so far in the clinicaltrails.gov database, pertaining to adult stem cell treatment for ED patients (Supplementary Table S1). Among those, four studies utilizing autologous adult stem cells were completed; however, no outcome data are available thus far. Six studies are ongoing and the status of two studies showed withdrawn, two studies are active but not recruiting and finally the status of two studies is unknown (Figure 4). Predominantly ADSCs were utilized for the most of these clinical trials, followed by bone marrow, umbilical cord, Wharton jelly, and placenta derived stem cells (Supplementary Table S1).

SVF represents a mixed cellular components made of a combination of ADSCs, endothelial precursor cells, endothelial cells, fibroblasts, macrophages, interstitial cells, smooth muscle cells, lymphocytes, pericytes, and pre-adipocytes among others [18–20]. Both cultured ADSCs and uncultured SVF were shown to exert similar effects in recovering penile erection in rat model of cavernosal nerve injury; however, SVF was superior to ADSCs in terms of histomorphometric changes like endothelial nitric-oxide synthase, smooth muscle/collagen ratio and von Willebrand factor expression [21]. Given the lack of concrete data on the effect of adipose tissue-derived SVF in treating ED in humans, we sought to determine the gene expression signature and signaling pathways of SVF fraction obtained from ED patients compared with healthy individuals to provide more insight into the suitability of SVF from ED patients for autologous transplantation and possibility to enhance their therapeutic efficacy.

Our data highlighted remarkable difference in the gene expression profile of SVF obtained from ED compared the SVF from healthy individuals. We observed significant enrichment in inflammatory and immune response in the up-regulated genes from ED patients, suggesting possible involvement of the immune system in this disease. Our data are in agreement with earlier studies correlating increased levels of inflammatory mediators (such as IL-6) in the circulation and the presence and severity of ED [22]. On the other hand, pathway analysis performed on the down-regulated genes in ED patient’s SVF revealed enrichment in MAPK, TGF-β, senescence, FAK, adipogenesis, androgen receptor, and EGF–EGFR signaling pathways. Our previous studies highlighted crucial role for several of the identified pathways in regulating MSC differentiation [23,24]. For instance, integrin-associated FAK regulates cell survival, adhesion, and stem cell maintenance and protects stem cells from apoptosis, detachment, and differentiation [25]. The MAPK pathway incorporates various signalling cascades, of which the Ras-Raf-Mek-ERK1/2 is one of the most dysregulated in cancer and plays important roles during normal physiology including cell proliferation, differentiation, survival, transformation, development, apoptosis inflammatory and other stress responses [26,27]. Down-regulation of MAPK and FAK pathways in ED patients indicates the poor physiological functions including angiogenesis and migration with inflammation and stress response.

Insufficiency of androgen disrupts cellular-signaling pathways and leading to abnormal erectile physiology [28], corroborating our findings on the down-regulation of androgen receptor pathway in SVF from ED patients. Moreover, well identified circadian clock pathway also been down-regulated in ED patients, this clock is synchronized by several environmental stimuli, mainly the light–dark (LD) cycle. Presently, sildenafil, vardenafil and tadalafil have been prescribed for ED to inhibit the phosphodiesterase (PDE5) and increase the low-intensity light-induced circadian responses [29]. In our data, we observed several of the ED patients to be obese or to have other metabolic disorders. Previous study demonstrated significant differences between subcutaneous and visceral/omental fractions of adipose tissue, which were associated with parameters such as BMI, BAI, and/or WHR values [30,31] Therefore, it is plausible that the differences obtained in current study are in part due to other pathological conditions associated with ED such as increase DM and obesity.

In conclusion, our data suggest the existence of multiple altered signaling pathways in the SVF from ED patients compare to healthy individuals, which could potentially play a role in the etiology of this disease with potential implications on autologous SVF transplant for ED patients. Weather these changes are drivers or consequences of ED remains to be elucidated. Therefore, therapeutic strategies targeting these pathways might provide novel therapeutic opportunity for ED patients.

## Supporting information

**Supplementary figure 1:** Laboratory findings in ED and healthy individuals included in current study. Biochemistry (**a**) and CBC (**b**) profile in ED (n=6) and healthy individuals (n=3) included in current study. Data are presented as mean ± S.D.

**Supplementary figure 2:** Illustration depicting the Focal adhesion pathway in SVF of ED patients

**Supplementary figure 3:** Illustration depicting the Angiogenesis pathway in SVF of ED patients.

**Supplementary figure 4:** Illustration depicting the inflammatory response pathway in SVF of ED patients.

**Supplementary Table 1:** Details of the current adult stem cell based clinical trial status for erectile dysfunction

## Abbreviations

ADSC: adipose derived stem cell
ED: erectile dysfunction
EGF: epidermal growth factor
EGFR: epidermal growth factor receptor
HDL: high density lipoprotein
LDL: low density lipoprotein
MAPK: mitogen-activated protein kinase
mRNA: messenger RNA
qRT-PCR: quantitative reverse transcription-PCR
SAT: subcutaneous adipose tissue
TGF-β: transforming growth factor beta

## References

[1] Shamloul R. and Ghanem H. (2013) Erectile dysfunction. Lancet 381, 153–165 23040455 10.1016/S0140-6736(12)60520-0

[2] Ponholzer A. (2005) Prevalence and risk factors for erectile dysfunction in 2869 men using a validated questionnaire. Eur. Urol. 47, 80–85, discussion 85-6 10.1016/j.eururo.2004.08.017 15582253

[3] Gatti A. (2009) Metabolic syndrome and erectile dysfunction among obese non-diabetic subjects. J. Endocrinol. Invest. 32, 542–545 10.1007/BF03346504 19494717

[4] Lue T.F. and Lee K.L. (2000) Pharmacotherapy for erectile dysfunction. Chin. Med. J. (Engl.) 113, 291–298 11775221

[5] Pederzoli F. (2018) Surgical factors associated with male and female sexual dysfunction after radical cystectomy: what do we know and how can we improve outcomes? Sex. Med. Rev., 6, 469–481 10.1016/j.sxmr.2017.11.003 29371143

[6] Reed-Maldonado A.B. and Lue T.F. (2016) The current status of stem-cell therapy in erectile dysfunction: a review. World J. Mens. Health 34, 155–164 10.5534/wjmh.2016.34.3.155 28053944 PMC5209555

[7] Bochinski D. (2004) The effect of neural embryonic stem cell therapy in a rat model of cavernosal nerve injury. BJU Int. 94, 904–909 10.1111/j.1464-410X.2003.05057.x 15476533

[8] Fall P.A. (2009) Apoptosis and effects of intracavernous bone marrow cell injection in a rat model of postprostatectomy erectile dysfunction. Eur. Urol. 56, 716–725 10.1016/j.eururo.2008.09.059 18922625

[9] Kendirci M. (2010) Transplantation of nonhematopoietic adult bone marrow stem/progenitor cells isolated by p75 nerve growth factor receptor into the penis rescues erectile function in a rat model of cavernous nerve injury. J. Urol. 184, 1560–1566 10.1016/j.juro.2010.05.088 20728109 PMC3014289

[10] Ying C. (2013) Effects of intracavernous injection of adipose-derived stem cells on cavernous nerve regeneration in a rat model. Cell. Mol. Neurobiol. 33, 233–240 10.1007/s10571-012-9890-7 23161147 PMC11497875

[11] Yiou R. (2016) Safety of intracavernous bone marrow-mononuclear cells for postradical prostatectomy erectile dysfunction: an open dose-escalation pilot study. Eur. Urol. 69, 988–991 10.1016/j.eururo.2015.09.026 26439886

[12] Haahr M.K. (2016) Safety and potential effect of a single intracavernous injection of autologous adipose-derived regenerative cells in patients with erectile dysfunction following radical prostatectomy: an open-label phase I clinical trial. EBioMedicine 5, 204–210 10.1016/j.ebiom.2016.01.024 27077129 PMC4816754

[13] Vishnubalaji R. (2012) Comparative investigation of the differentiation capability of bone-marrow- and adipose-derived mesenchymal stem cells by qualitative and quantitative analysis. Cell Tissue Res. 347, 419–427 10.1007/s00441-011-1306-3 22287041

[14] Vishnubalaji R. (2018) Molecular profiling of ALDH1(+) colorectal cancer stem cells reveals preferential activation of MAPK, FAK, and oxidative stress pro-survival signalling pathways. Oncotarget 9, 13551–13564 10.18632/oncotarget.24420 29568377 PMC5862598

[15] Vishnubalaji R. (2015) Genome-wide mRNA and miRNA expression profiling reveal multiple regulatory networks in colorectal cancer. Cell Death Dis. 6, e1614 10.1038/cddis.2014.556 25611389 PMC4669754

[16] Phinney D.G. and Prockop D.J. (2007) Concise review: mesenchymal stem/multipotent stromal cells: the state of transdifferentiation and modes of tissue repair–current views. Stem Cells 25, 2896–2902 10.1634/stemcells.2007-0637 17901396

[17] Vishnubalaji R. (2012) In vitro differentiation of human skin-derived multipotent stromal cells into putative endothelial-like cells. BMC Dev. Biol. 12, 7 10.1186/1471-213X-12-7 22280443 PMC3280173

[18] Rhee S.C. (2011) In vivo evaluation of mixtures of uncultured freshly isolated adipose-derived stem cells and demineralized bone matrix for bone regeneration in a rat critically sized calvarial defect model. Stem Cells Dev. 20, 233–242 10.1089/scd.2009.0525 20528145

[19] Bora P. and Majumdar A.S. (2017) Adipose tissue-derived stromal vascular fraction in regenerative medicine: a brief review on biology and translation. Stem Cell Res. Ther. 8, 145 10.1186/s13287-017-0598-y 28619097 PMC5472998

[20] Bourin P. (2013) Stromal cells from the adipose tissue-derived stromal vascular fraction and culture expanded adipose tissue-derived stromal/stem cells: a joint statement of the International Federation for Adipose Therapeutics and Science (IFATS) and the International Society for Cellular Therapy (ISCT). Cytotherapy 15, 641–648 10.1016/j.jcyt.2013.02.006 23570660 PMC3979435

[21] You D. (2015) Comparative study of autologous stromal vascular fraction and adipose-derived stem cells for erectile function recovery in a rat model of cavernous nerve injury. Stem Cells Transl. Med. 4, 351–358 10.5966/sctm.2014-0161 25792486 PMC4367505

[22] Vlachopoulos C. (2007) Inflammation, metabolic syndrome, erectile dysfunction, and coronary artery disease: common links. Eur. Urol. 52, 1590–1600 10.1016/j.eururo.2007.08.004 17707576

[23] Ali D. (2018) Multiple intracellular signaling pathways orchestrate adipocytic differentiation of human bone marrow stromal stem cells. Biosci. Rep. 38, BSR20171252, 1-10. 10.1042/BSR20171252 29298881 PMC5789155

[24] Ali D. (2016) Epigenetic library screen identifies abexinostat as novel regulator of adipocytic and osteoblastic differentiation of human skeletal (mesenchymal) stem cells. Stem Cells Transl. Med. 5, 1036–1047 10.5966/sctm.2015-0331 27194745 PMC4954455

[25] Vitillo L. and Kimber S.J. (2017) Integrin and FAK regulation of human pluripotent stem cells. Curr. Stem Cell Rep. 3, 358–365 10.1007/s40778-017-0100-x 29177133 PMC5683053

[26] Zhang W. and Liu H.T. (2002) MAPK signal pathways in the regulation of cell proliferation in mammalian cells. Cell Res. 12, 9–18 10.1038/sj.cr.7290105 11942415

[27] Dhillon A.S. (2007) MAP kinase signalling pathways in cancer. Oncogene 26, 3279–3290 10.1038/sj.onc.1210421 17496922

[28] Traish A.M., Goldstein I. and Kim N.N. (2007) Testosterone and erectile function: from basic research to a new clinical paradigm for managing men with androgen insufficiency and erectile dysfunction. Eur. Urol. 52, 54–70 10.1016/j.eururo.2007.02.034 17329016 PMC2562639

[29] Plano S.A. (2012) cGMP-phosphodiesterase inhibition enhances photic responses and synchronization of the biological circadian clock in rodents. PLoS One 7, e37121 10.1371/journal.pone.0037121 22590651 PMC3349644

[30] Blogowski W. (2013) Clinical analysis of selected complement-derived molecules in human adipose tissue. J. Transl. Med. 11, 11 10.1186/1479-5876-11-11 23302473 PMC3570347

[31] Blogowski W. (2012) Clinical analysis of systemic and adipose tissue levels of selected hormones/adipokines and stromal-derived factor-1. J. Biol. Regul. Homeost. Agents 26, 607–615 23241111

