# Supplementary material for: Whole genome mRNA expression profiling revealed multiple deregulated pathways in stromal vascular fraction from erectile dysfunction patients
Source: Biosci Rep. 2018 Nov 23;38(6):BSR20181015. doi: 10.1042/BSR20181015 (PMC6250806; doi:10.1042/BSR20181015)
Supplement: Supplementary file 1 [file bsr20181015_Supp1.pdf]

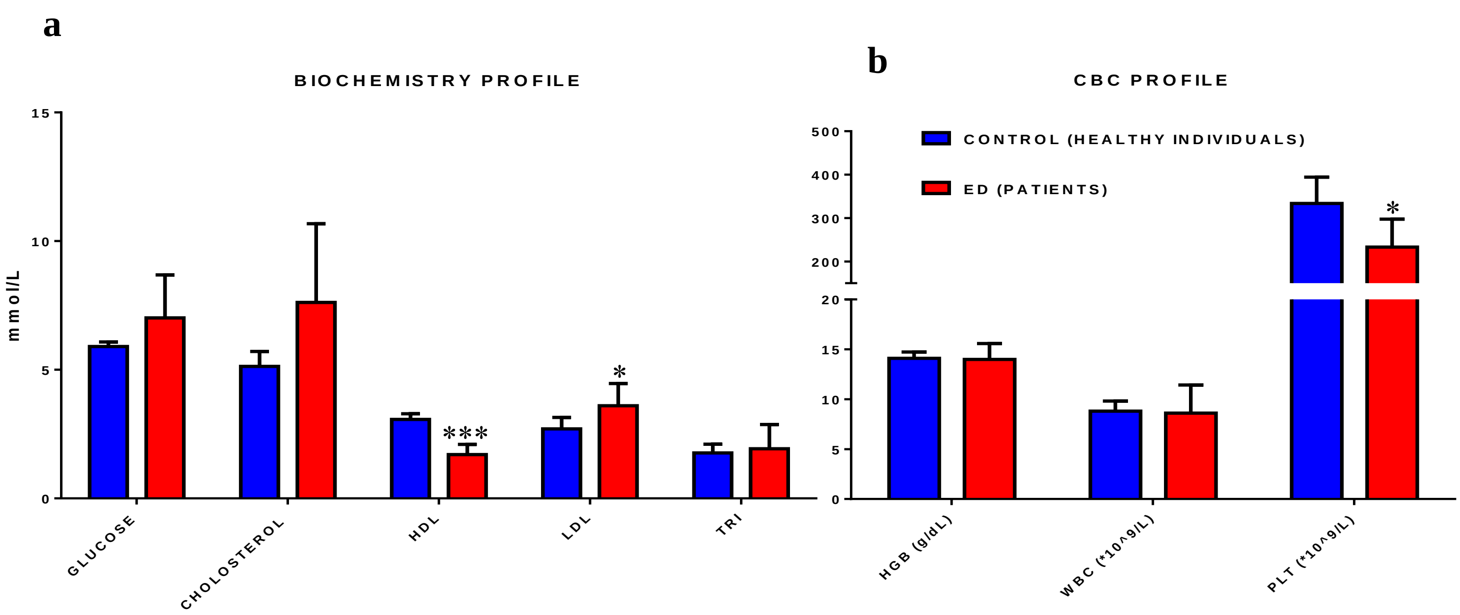

Supplementary figure 1. Laboratory findings in ED and healthy individuals included in current study. Biochemistry **(a)** and CBC **(b)** profile in ED (n=6) and healthy individuals (n=3) included in current study. Data are presented as mean ± S.D.

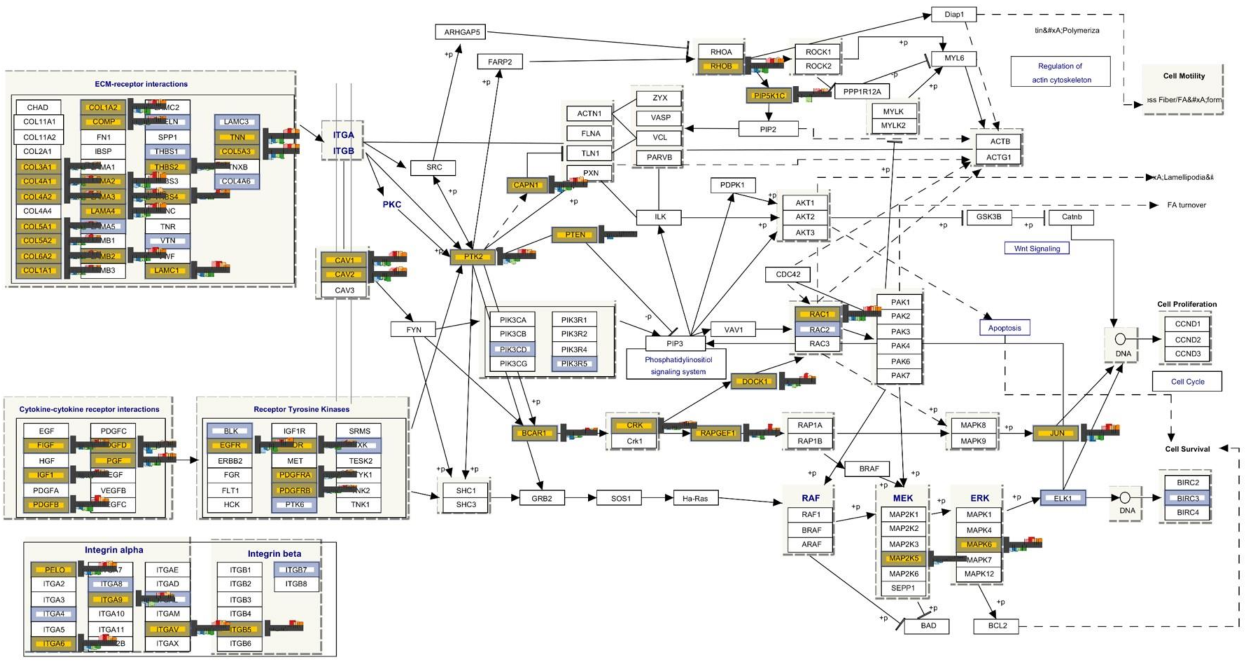

Supplementary figure 2. Illustration depicting the Focal adhesion pathway in SVF of ED patients

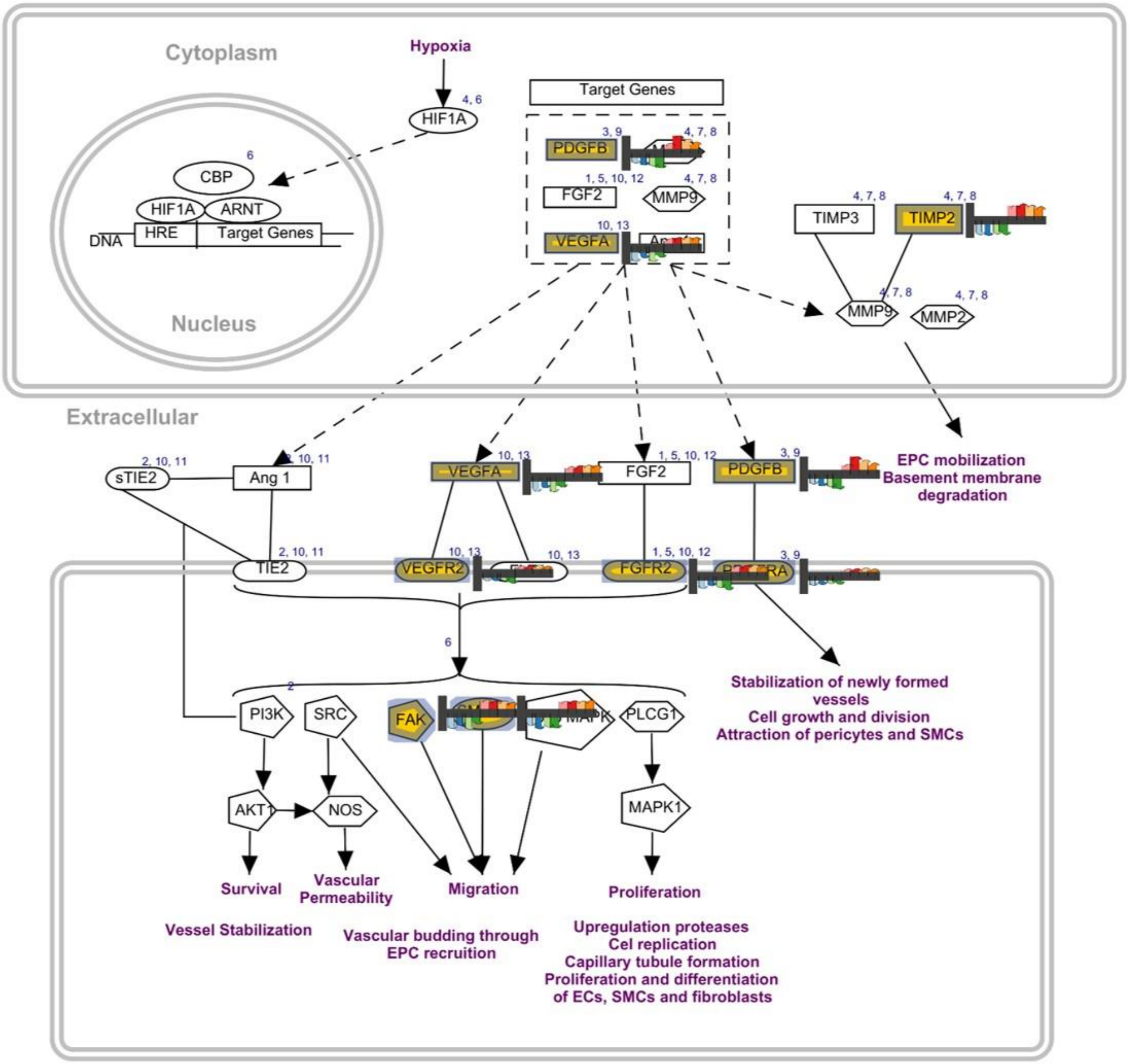

Supplementary figure 3. Illustration depicting the Angiogenesis pathway in SVF of ED patients.

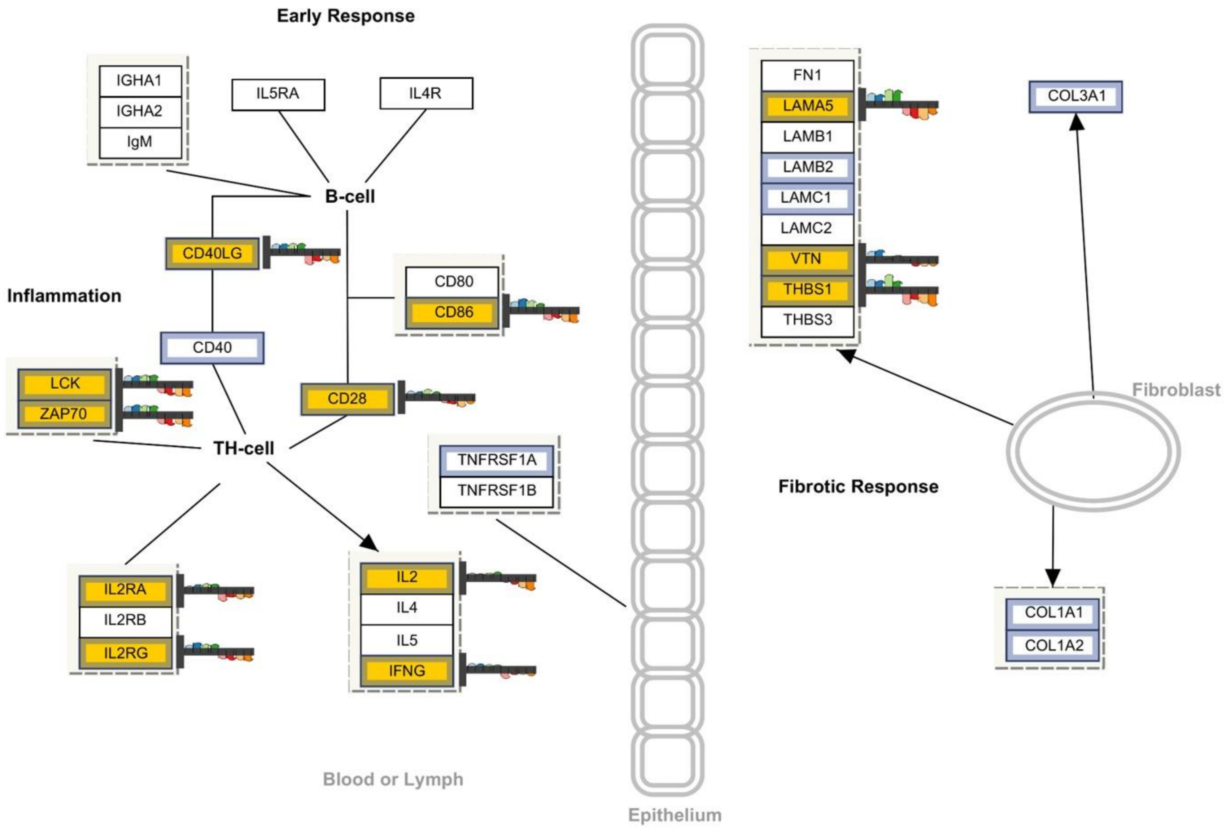

Supplementary figure 4. Illustration depicting the inflammatory response pathway in SVF of ED patients
